# Peritoneal M2 macrophage transplantation as a potential cell therapy for enhancing renal repair in acute kidney injury

**DOI:** 10.1111/jcmm.15005

**Published:** 2020-01-31

**Authors:** Ruiwen Mao, Chengshi Wang, Fuping Zhang, Meng Zhao, Shuyun Liu, Guangneng Liao, Lan Li, Younan Chen, Jingqiu Cheng, Jingping Liu, Yanrong Lu

**Affiliations:** ^1^ Key Laboratory of Transplant Engineering and Immunology Regenerative Medicine Research Center West China Hospital Sichuan University Chengdu China; ^2^ West China School of Nursing West China Hospital Sichuan University Chengdu China

**Keywords:** acute kidney injury, cell therapy, inflammation, peritoneal M2 macrophage, renal repair

## Abstract

Acute kidney injury (AKI) is a clinical condition that is associated with high morbidity and mortality. Inflammation is reported to play a key role in AKI. Although the M2 macrophages exhibit antimicrobial and anti‐inflammatory activities, their therapeutic potential has not been evaluated for AKI. This study aimed to investigate the protective effect of peritoneal M2 macrophage transplantation on AKI in mice. The macrophages were isolated from peritoneal dialysates of mice. The macrophages were induced to undergo M2 polarization using interleukin (IL)‐4/IL‐13. AKI was induced in mice by restoring the blood supply after bilateral renal artery occlusion for 30 minutes. The macrophages were injected into the renal cortex of mice. The changes in renal function, inflammation and tubular proliferation were measured. The M2 macrophages were co‐cultured with the mouse primary proximal tubular epithelial cells (PTECs) under hypoxia/reoxygenation conditions in vitro. The PTEC apoptosis and proliferation were analysed. The peritoneal M2 macrophages effectively alleviated the renal injury and inflammatory response in mice with ischaemia‐reperfusion injury (IRI) and promoted the PTEC proliferation in vivo and in vitro. These results indicated that the peritoneal M2 macrophages ameliorated AKI by decreasing inflammatory response and promoting PTEC proliferation. Hence, the peritoneal M2 macrophage transplantation can serve as a potential cell therapy for renal diseases.

## 1 INTRODUCTION

Globally, acute kidney injury (AKI) is a serious clinical condition associated with high morbidity and mortality. The leading cause of AKI is ischaemia‐reperfusion injury (IRI), which usually occurs during trauma, surgical resection and kidney transplantation.^1^, ^2^, ^3^ Several studies have indicated that inflammatory response plays a key role in AKI.^2^ After AKI, the renal tubular epithelial cells undergo massive necrosis or apoptosis, which results in a rapid decline in renal function within a short period.^3^ Currently, the only clinical treatment for AKI is supportive therapy. There are no drugs (therapeutic or preventive) that have been approved for the clinical treatment of AKI.^4^ Therefore, there is a need to devise an effective therapy for AKI that can alleviate inflammatory response and promote renal regeneration.

The macrophages are multifunctional and heterogeneous innate immune cells, which play important roles in regulating inflammation.^5^, ^6^ The mature macrophages can alter their phenotypes and undergo functional polarization in response to environmental signals. There are two well‐known macrophage polarization programs: classically activated (M1) macrophages, which exhibit pro‐inflammatory properties and alternatively activated (M2) macrophages, which are involved in tissue repair and resolution of inflammation.^7^, ^8^, ^9^ The macrophages exhibit a spectrum of activated phenotypes rather than discrete stable subpopulations. The M1 and M2 macrophages represent two extreme phenotypic spectra with a continuum of intermediate phenotypes between M1 and M2 phenotypes.^10^ The macrophages are reported to have pathogenic roles in human kidney diseases. After AKI, the neutrophils and natural killer cells are recruited to the damaged renal tissue within hours, which is followed by the infiltration of inflammatory monocytes.^11^ These inflammatory monocytes are then polarized into M1 macrophages, which is mediated by various pro‐inflammatory mediators, such as interleukin (IL)‐1β, IL‐6, tumour necrosis factor (TNF)‐α and reactive oxygen species (ROS), to exacerbate early tubular injury.^8^ However, during the later recovery phase, the macrophages switch to the M2 phenotype, which is involved in anti‐inflammatory activities and tissue repair and remodelling. This indicated a protective role of M2 macrophages in tissue recovery.^8^, ^12^ The M2 phenotype can be induced ex vivo or in vivo via IL‐10 and transforming growth factor (TGF)‐β stimulation for treating kidney diseases.^13^ The type 2 innate lymphoid cells (ILC2s), which are a recently described innate immune cell population that can be activated by IL‐33 or IL‐25, also contribute to M2 polarization of macrophages through secretion of various type 2 cytokines, such as IL‐4 and IL‐13.^14^, ^15^, ^16^

Recent studies have demonstrated that macrophages can be potentially used in cell therapies to induce immune tolerance to transplantation and to promote wound healing.^17^, ^18^ Cao Q et al^13^, ^19^ polarized the splenic macrophages to the M2 phenotype, which effectively reduced renal injury, by adoptive transfer of M2 macrophages to experimental models of adriamycin‐induced kidney disease. Lee et al^12^ demonstrated that the anti‐inflammatory M2 macrophages promoted kidney repair after AKI. The CD11c^+^ cells are reported to be involved in the recovery process after AKI. The depletion of macrophages by liposome clodronate treatment during the recovery phase was associated with persistent tubular damage and inflammation.^20^ In an adriamycin nephrosis (AN) model, both M2a and M2c macrophages were reported to reduce renal inflammation.^21^ However, it is difficult to obtain a large number of macrophages (approximately 10^6^ cells/kg patient's weight) from the blood of the patients. Moreover, unlike T cells, macrophages are differentiated cells that cannot be expanded in vitro to meet the demand of cell therapy.^22^ Therefore, there is a need to explore alternative sources of macrophages to meet the requirement of cell therapy.

Previous studies have reported that large numbers of monocytes/macrophages could be retrieved from effluent dialysis bags of patients undergoing peritoneal dialysis (PD). Approximately 40% of these cells are monocytes/macrophages.^23^, ^24^, ^25^, ^26^ The macrophages from peritoneal dialysates are a rich source of mononuclear phagocytes that can be used for cell therapy. The phenotype of both human and mouse monocytes/macrophages could be polarized to the regulatory M2 phenotype by IL‐4/IL‐13.^27^ The peritoneal M2 macrophages derived from peritoneal dialysates of mice markedly reduced the inflammatory infiltrates and kidney injury, while the peritoneal M1 macrophages exacerbated kidney injury in mice with AN.^19^ However, the mechanism underlying peritoneal macrophage‐mediated renal repair is not completely understood.

In this study, we aimed to evaluate the renal protective role of peritoneal M2 macrophage transplantation in hypoxia/reoxygenation (H/R)‐induced renal proximal tubular epithelial cell (PTEC) injury and IRI‐induced AKI in mice and evaluated the potential underlying mechanism.

## 2 MATERIALS AND METHODS

### 2.1 Mouse PD model

The mouse PD model was established as previously described.^28^ The C57BL/6 mice were anaesthetized, and a silicon catheter (22 G) was inserted percutaneously into the left lower quadrant of the peritoneal cavity via a subcutaneous tunnel. The peritoneal fluid was drained, and the cells in the dialysate were collected every day for 15 days. The peritoneal macrophages from peritoneal dialysate were used in the following experiments.

### 2.2 Macrophage isolation and M2 polarization

The macrophages derived from peritoneal dialysate of C57BL/6 mice were enriched using the CD11b^+^ microbeads (Miltenyi Biotec). The enriched macrophages were cultured in the RPMI 1640 medium supplemented with 10% foetal bovine serum (FBS, Thermo Fisher Scientific), penicillin (50 U/mL), streptomycin (50 µg/mL) and macrophage colony‐stimulating factor (M‐CSF; 10 ng/mL, Invitrogen) overnight. The purity of macrophages was evaluated by flow cytometric analysis, which revealed >95% purity. The M0 macrophages were cultured for 48 hours in the presence of M‐CSF and IL‐4/IL‐13 (20 ng/mL each, Invitrogen) to induce the M2 polarization.

### 2.3 Isolation and culturing of PTECs

The mouse PTECs were isolated as described previously.^12^ Briefly, the mice were anaesthetized with pentobarbital sodium (50 mg/kg). The capsule and medulla were excised using a sharp blade. The renal cortex, which was cut into 1 mm^3^ pieces, was incubated with type IV collagenase (1 mg/mL, Roche) and DNase I (100 µg/mL, Roche) solution at 37°C for 40 minutes. The enzyme‐containing solution was then removed. The digested cells were filtered through the 80‐mesh and 100‐mesh strainers. The cells were cultured in DME/F12 (Hyclone Laboratories) supplemented with 10% FBS, 50 U/mL penicillin and 50 µg/mL streptomycin in a humidified atmosphere at 37°C and 5% CO_2_. The PTECs were identified by immunofluorescence (IF) staining using the specific anti‐cytokeratin 18 antibodies (1:1000, Servicebio, Sino Biological Inc).

### 2.4 Cell co‐culture and treatment

To induce H/R injury, the PTECs were subjected to hypoxia in a hypoxic chamber (≤1% O_2_, 5% CO_2_, 94% N_2_) at 37°C for 24 hours, followed by reoxygenation (21% O_2_, 5% CO_2_, 74% N_2_) for 2 hours. The PTECs were co‐cultured with M0 or M2 macrophages under H/R conditions. For co‐culture experiments, the M0 or M2 macrophages were seeded in a transwell insert at a density of 1.0 × 10^5^/cm^2^ and cultured in the RPMI 1640 medium for 12 hours. The M0 or M2 macrophages were washed thrice with phosphate buffer solution (PBS) and then co‐cultured with PTECs (ratio 1:1) in glucose and FBS‐free Dulbecco's modified Eagle's medium (DMEM).

### 2.5 Cell viability assay

The PTECs were seeded in a 96‐well plate and co‐cultured with M2 macrophages under H/R conditions. Next, the cells were incubated with CCK8 solution (Dojindo) for 2 hours at 37°C. The absorbance of the samples was measured at 450 nm using a microplate reader (BioTek Instruments Inc). The cell viability was calculated by normalizing the optical density of the experimental group to that of the control group.

### 2.6 Quantitative real‐time polymerase chain reaction (qPCR)

Total RNA was isolated from the renal tissues using Trizol (Gibco, Life Technologies), following the manufacturer's instructions. The isolated RNA was quantified using the NanoDrop 2000 spectrophotometer (Thermo Fisher Scientific Inc). The RNA was reverse transcribed to cDNA using the iScript cDNA Synthesis kit (Bio‐Rad). The primer sequences are listed in Table S1. The qPCR analysis was performed on a CFX96 Real‐Time PCR Detection System (Bio‐Rad) with SYBR green supermix (SsoFast EvaGreen, Bio‐Rad). The relative change in the expression of target genes mRNA were calculated using the 2^‐ΔΔCT^ method.

### 2.7 Western blotting analysis

The cells and mouse renal tissues were homogenized in lysis buffer containing phenylmethanesulphonyl fluoride (PMSF). The protein concentrations were measured using the bicinchoninic acid (BCA) protein assay kit (Beyotime Biotechnology). Equal amount of protein was subjected to sodium dodecyl sulphate‐polyacrylamide gel electrophoresis (SDS‐PAGE) using 10% gel. The resolved proteins were then transferred to a polyvinylidene difluoride membrane (0.22 µm PVDF, Merck Millipore). The membrane was incubated with 5% nonfat milk for 1 hour for blocking. Next, the membrane was probed with the primary antibodies against NLRP3 (1:500, Proteintech), IL‐1β (1:1000, Abcam), Smad7 (1:200, Santa Cruz Biotechnology, Inc), CyclinD1 (1:200, Servicebio) and β‐Actin (1:10000, Abclonal) overnight at 4°C. The membrane was then incubated with the horseradish peroxidase (HRP)‐conjugated secondary antibodies (1:2000, Santa Cruz Biotechnology, Inc). The protein bands were visualized using the enhanced chemiluminescence (ECL) reagents in the Molecular Imager Gel Doc XR System (Bio‐Rad). The blots were subjected to densitometric analysis using the Image J software (NIH). The protein expression was normalized to the expression of β‐Actin.

### 2.8 Flow cytometric analysis

For fluorescence‐activated cell sorting (FACS) analysis of mouse cells, the single‐cell suspensions from kidney samples were incubated with the anti‐CD32/16 antibody to block the Fc receptors, followed by incubation with antibodies against CD45.2, CD11c, F4/80, CD206, CD3, CD4 and CD8 (all from BD Biosciences). The cells were analysed in a flow cytometer (Beckman Coulter).

### 2.9 Cell apoptosis assay

Cell apoptosis was analysed using the Annexin V‐FITC/PI staining kit (Roche Applied Science). The treated cells were collected and labelled for 15 minutes with annexin V and propidium iodide (PI). The apoptotic cells were detected in a flow cytometer (Beckman Coulter).

### 2.10 Cell cycle analysis

The treated cells were harvested, washed with cold PBS and fixed in 70% ethanol. Next, the cell samples were washed, resuspended in PBS and incubated with PI (100 µg/mL, Sigma‐Aldrich) and RNase (200 µg/mL, Sigma‐Aldrich) solution at 34‐37°C for 30 minutes in the dark. The stained cell samples were analysed using the FC500 flow cytometer (Beckman Coulter). The percentage of cells in each cell cycle phase was calculated. All cell samples were measured in triplicates. The proliferation index was calculated as follows: proliferation index = (S + G2/M) ÷ (G1 + S + G2/M) (S, G1,G2 and M, respectively, indicate the percentage of cells contained in each period).

### 2.11 IF staining

The cells were washed in PBS, fixed with 4% paraformaldehyde in PBS for 10 minutes, permeabilized with 0.1% Triton X‐100 in PBS for 10 minutes, and blocked in 1% bovine serum albumin (BSA) in PBS for 30 minutes. The cells were incubated with the primary antibodies against F4/80, CD206 and iNOS (1:300; Santa Cruz Biotechnology) for 1 hour at 34‐37°C. The cells were then incubated with the secondary antibodies (1:500) for 1 hour. The cells were washed with PBS and incubated with 4′,6‐diamidino‐2‐phenylindole (DAPI; Sigma‐Aldrich) for 5 minutes. Next, the cells were mounted with Fluoromount‐G™ (eBioscience) and observed under a fluorescent microscope.

### 2.12 IRI mice model and cell treatment

All animal experiments were approved and conducted in accordance with the guidelines of Animal Care and Use Committee of Sichuan University. Adult male C57BL/6 mice (25‐30 g) were purchased from the Experimental Animal Center of Sichuan University (Chengdu, China). The animals were housed in pairs of two in cages with controlled temperature (20‐22°C), humidity (40%‐60%) and 12‐hours light/dark cycle. The animals were fed with standard chow and sterile water ad libitum. The health of the animals was monitored every day. None of the animals exhibited severe signs of illness or died due to the experimental treatment. The mice were anaesthetized with an intraperitoneal injection of pentobarbital (50 mg/kg, Merck) and placed on a warm pad to maintain the body temperature at 34.5‐37°C. The mice were then randomized to sham or IRI operation groups. The renal pedicles were exposed by flank incisions for bilateral clamping to induce ischaemia for 30 minutes. The clamps were then released for reperfusion. The colour change of kidneys was observed to visually monitor ischaemia and reperfusion. The sham control mice underwent the same operation without renal pedicle clamping. The M0 or M2 macrophages (1 × 10^6^ cells in 50 µL PBS) were injected into the bilateral renal cortex at 10 minutes post‐reperfusion. The mice in each group were sacrificed by overdose of anaesthesia at day 3 post‐surgery. The renal tissue and serum were collected for further tests.

### 2.13 Biochemical measurement

The serum creatinine (CREA), blood urea nitrogen (BUN) and uric acid (UA) levels in the mice were analysed on an Automatic Biochemistry Analyzer (Cobas Integra 400 plus, Roche) using appropriate kits.

### 2.14 Histological examination

The renal cross sections were subjected to haematoxylin and eosin (H&E) and periodic acid‐Schiff (PAS) staining. The tissue damage was examined and scored based on the percentage of damaged tubules as follows: 0, no damage; 1, <25%; 2, 25%‐50%; 3, 50%‐75%; 4, >75%. The samples were blinded to the assessor. The mean tubular injury scores for each mouse represented the average score of the 20 fields examined.^28^, ^29^, ^30^ The expression levels of CD3, Ki67 and CDK (Santa Cruz Biotechnology) in the kidney tissues were evaluated by immunohistochemical (IHC) staining. The digital images of stained sections were captured under a light microscope (Zeiss Imager A2).

### 2.15 Statistical analysis

Descriptive statistics were presented as mean ± standard error of mean (SEM) and analysed in the SPSS software (version 11.5, IBM Corp). All data were analysed using the Kolmogorov‐Smirnov test to confirm the normal distribution. Student's *t* test was employed for comparisons between two groups. One‐way analysis of variance (ANOVA) followed by Tukey's ad hoc test was used for multiple comparisons. All data were analysed using the two‐tailed test. The difference was considered statistically significant when the *P*‐value was <.05.

## 3 RESULTS

### 3.1 Induction of peritoneal M2 macrophage in vitro

**Figure 1:**
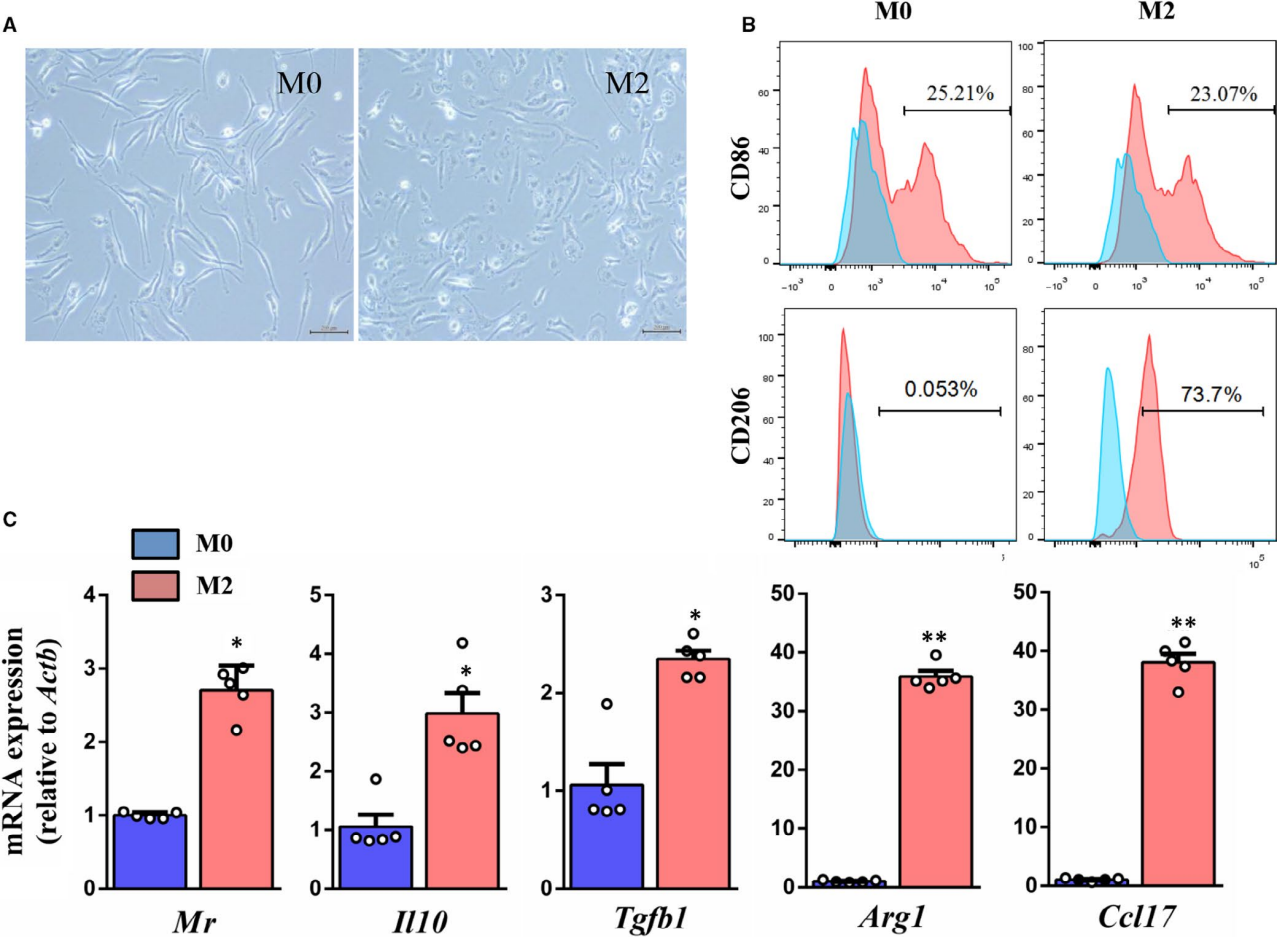
Isolation and validation of mice peritoneal macrophages. A, Microscopic views of M0 and M2 macrophages (scale bar = 200 µm). B, Flow cytometric analysis of macrophage markers CD86 and CD206. C, Real‐time PCR analysis of M2 macrophages markers Mr, Il10, Tgfb1, Arg1 and Ccl17 at mRNA level. *P < .05, **P < .01 vs M0 (n = 5)

The phenotype of mononuclear cells obtained from peritoneal dialysates polarized to M0 macrophages upon induction with M‐CSF and then to M2 macrophages upon induction with the combination of IL‐4 and IL‐13. As shown in Figure 1A, the M0 macrophages exhibited elongated shape, while the M2 macrophages exhibited round, oval or spindle shape. The FACS analysis revealed that the number of CD86^+^ M0 macrophages was similar to the number of CD86^+^ M2 macrophages. Additionally, the number of CD206^+^ M2 macrophages was markedly higher than the number of CD206^+^ M0 macrophages (Figure 1B). Compared with the M0 macrophages, the M2 macrophages exhibited higher mRNA expression levels of mannose receptor (*Mr/Mrc1*), *Il10*, *Tgfb1*, *Arg1* and *Ccl17* (Figure 1C).

### 3.2 Peritoneal M2 macrophages attenuated renal injury in IRI mice

**Figure 2:**
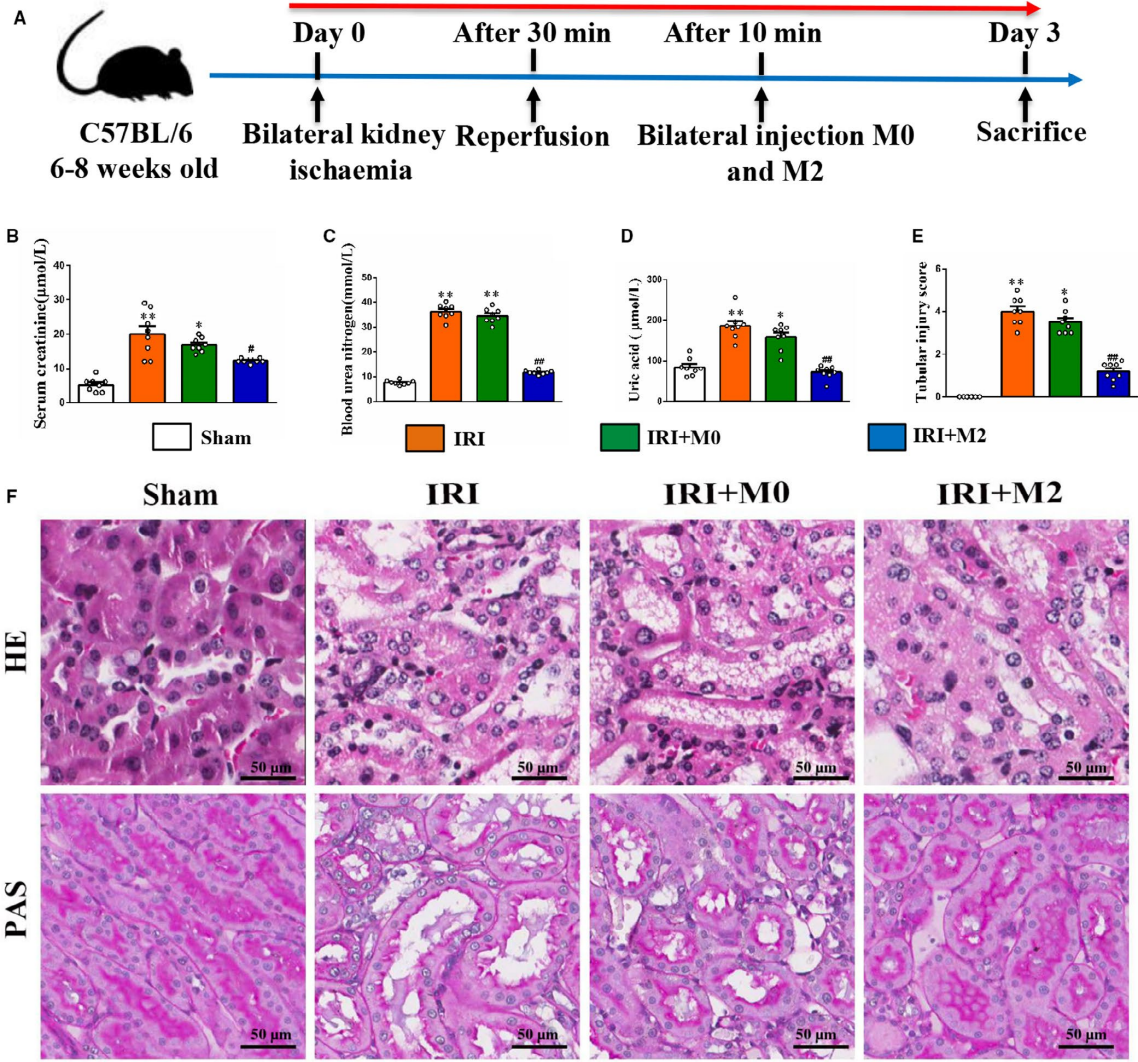
M2 macrophages improved kidney function in IRI mice. A, The detailed experimental scheme of animal study. B, Serum CREA (C) BUN and (D) uric acid (UA) at day 3 after surgery. (E) Representative micrographs of renal H&E and PAS staining (scale bar = 50 µm) from mice of different groups. F, The percentage of necrotic tubules in mice at day 3 after IRI. *P < .05, **P < .01 vs Sham. #*P* < .05, ##*P* < .01 vs IRI (n = 8)

To determine the role of M2 macrophages in renal injury, we adoptively transplanted peritoneal macrophages into the kidneys of IRI mice. The detailed experimental scheme of animal study is shown in Figure 2A. The levels of renal function parameters, such as BUN, CREA and UA in the IRI mice (IRI group) and M0 macrophage‐transplanted IRI mice (IRI + M0 group), were higher than those in the sham group. The levels of BUN, CREA and UA in the M2 macrophage‐transplanted IRI mice (IRI + M2 group) were significantly lower than those in the sham group (Figure 2B‐D). There was no significant difference between the levels of BUN, CREA and UA in the control group and PBS group (Figure S4A). The IRI group exhibited enhanced renal lesions, including tubular hypertrophy, loss of brush border, vacuolar degeneration and necrotic tubules when compared to the sham group (Figure 2E‐F). This indicated that the M2 macrophage transplantation decreased renal lesions in IRI mice, whereas M0 macrophage transplantation had no effect on renal injury in IRI mice.

### 3.3 Peritoneal M2 macrophages attenuated renal inflammatory response in IRI mice

**Figure 3:**
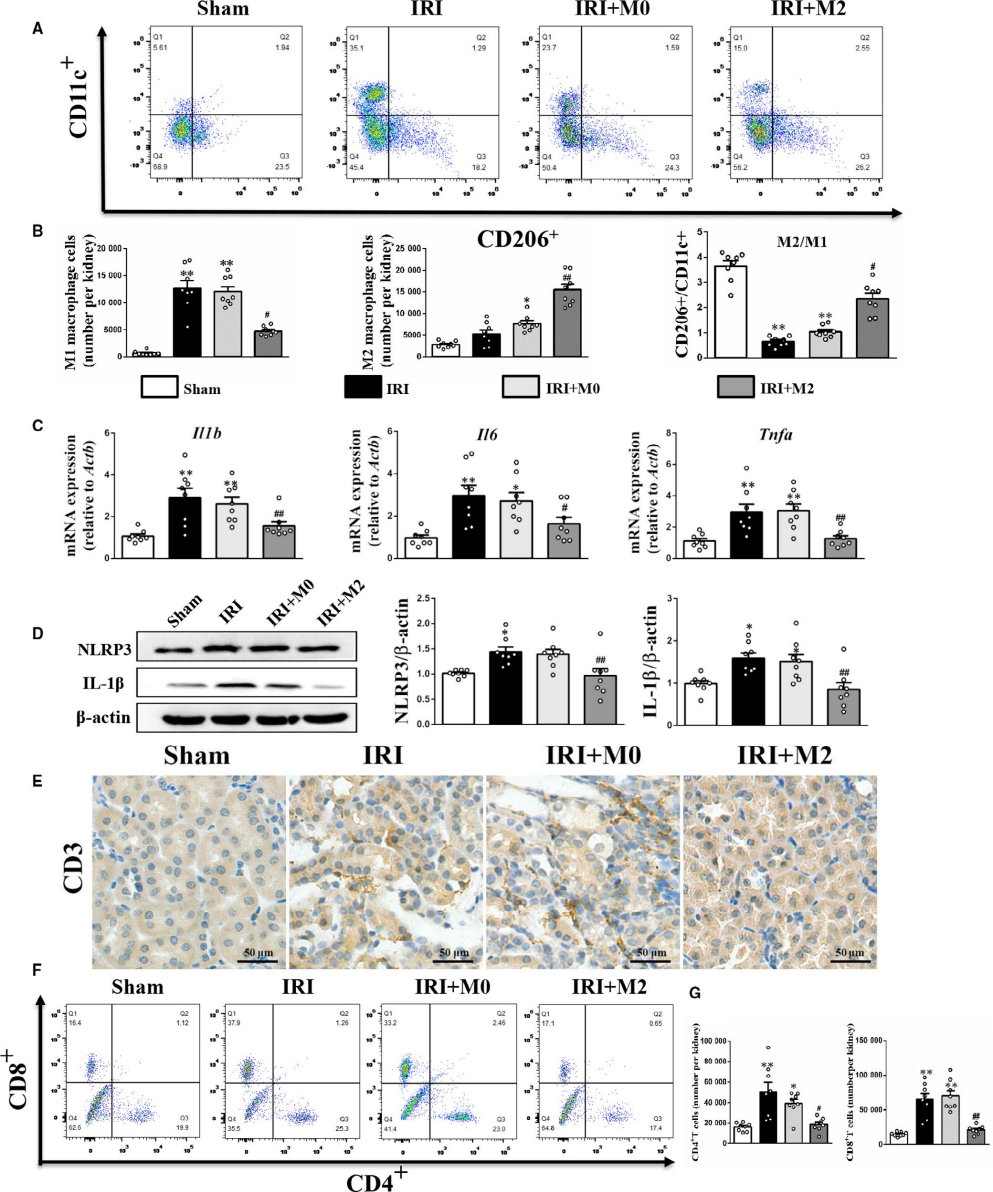
M2 macrophages reduced inflammatory response in kidneys after IRI. A, Flow cytometry analysis of M2 and M1 macrophages in kidney at day 3 after IRI. B, The absolute number of M1 and M2 macrophages in mice per kidney tissue, and quantification analysis of M2/M1 ratio in kidney. C, Real‐time PCR analysis of Il1b, Il6 and Tnfα mRNA level at day 3 after IRI. D, Western blot analysis of NLRP3 and IL‐1β protein level at day 3 after IRI. E, IHC staining for CD3 at day 3 after IRI (Scale bar = 50 µm). F, Flow cytometry analysis of CD4 + and CD8 + T cells in kidney at day 3 after IRI. G, The absolute number of CD4 + and CD8 + T cells in mice per kidney tissue. *P < .05, **P < .01 vs Sham. #*P* < .05, ##*P* < .01 vs IRI (n = 8)

**Figure 4:**
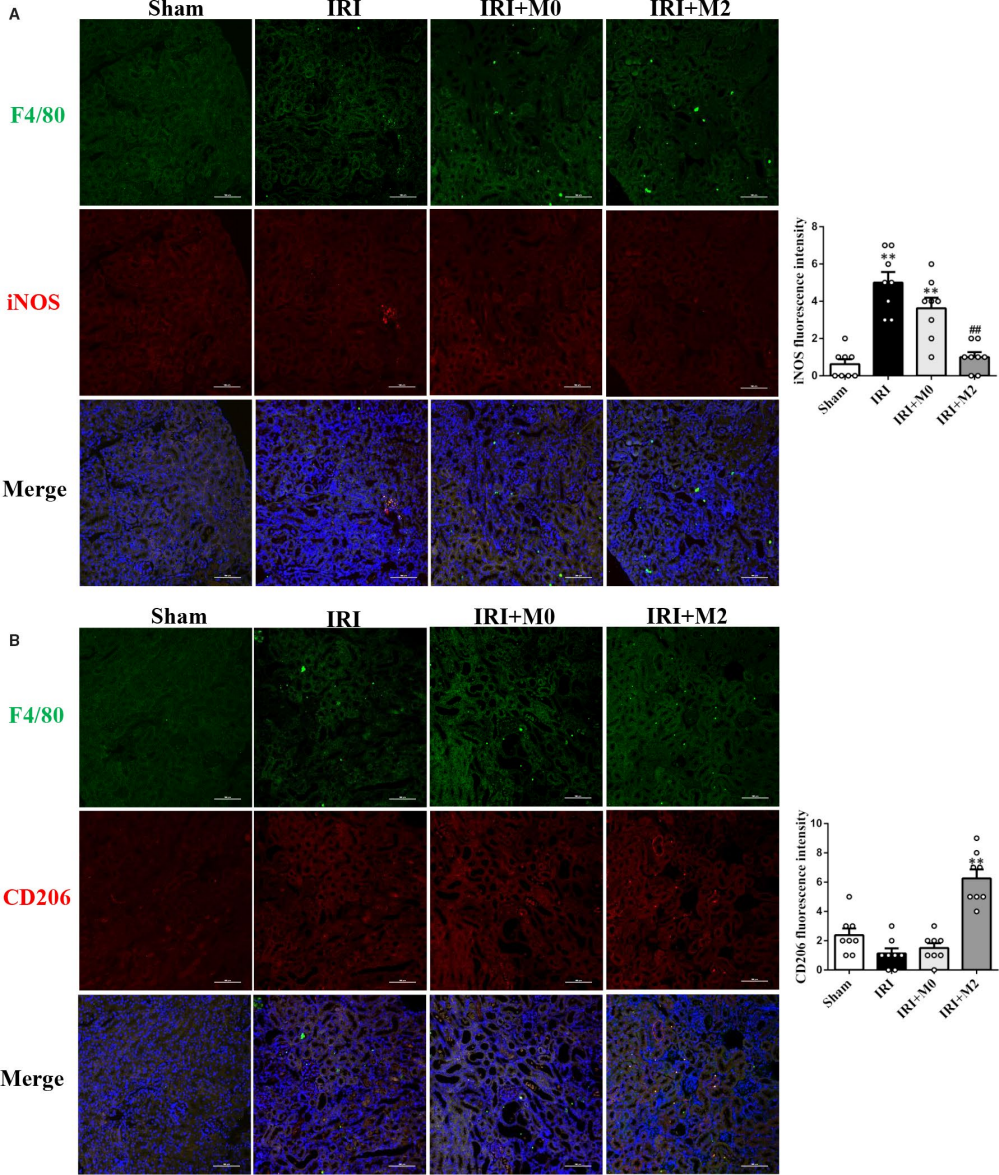
Transfused peritoneal M2 reduced renal inflammation in IRI mice. A, IF staining of M1 and (B) M2 macrophages in kidneys at day 3 after IRI. (Scale bar = 100 µm) *P < .05, **P < .01 vs Sham. #*P* < .05, ##*P* < .01 vs IRI (n = 8)

The macrophages were isolated from kidneys of the IRI group at day 3 post‐transplantation. The flow cytometric analysis revealed that the number of CD206^+^/CD11c^+^ M2 macrophages in the kidneys of the IRI + M2 group was significantly higher than that in the IRI and IRI + M0 groups (Figure 3A‐B). The flow cytometry gating strategy is shown in Figure S3A. The quantitative analysis of M1 and M2 macrophages also revealed that the M1 macrophages were abundant in the kidney of IRI and IRI + M0 groups, while the M2 macrophages were significantly abundant in the kidney of IRI + M2 group (Figure 3B). The qPCR and Western blotting analyses revealed that the IRI group exhibited enhanced expression of pro‐inflammatory factors, including *Il1b*, *Il6* and *Tnfα* mRNA and NLRP3 and IL‐1β protein when compared to the sham group. Contrastingly, the IRI + M2 group exhibited significantly down‐regulated expression of these cytokines (Figure 3C‐D). There was no significant difference between the mRNA levels of cytokines (*Il1b*, *Il6* and *Tnfα*) in the control group and PBS group (Figure S4B). The original Western blot figures are shown in Figure S2A. The number of CD3^+^ T cells in the kidney of IRI group was significantly higher than that in the kidney of IRI + M2 group. There was no difference in the T‐cell infiltration between the IRI and IRI + M0 groups (Figure 3E). The flow cytometric analysis revealed that the number of CD4^+^ T cells and CD8^+^ T cells in the kidneys of IRI group was significantly higher than that in the kidneys of IRI + M2 group (Figure 3F‐G). The flow cytometry gating strategy is shown in Figure S3B. To further validate the subtypes of macrophages, the M1 cells were subjected to co‐immunostaining with F4/80 and iNOS, while the M2 cells were subjected to co‐immunostaining with F4/80 and CD206. The IRI and IRI + M0 groups exhibited increased abundance of M1 macrophages, whereas the IRI + M2 group exhibited increased abundance of M2 macrophages (Figure 4A‐B).

### 3.4 Peritoneal M2 macrophages promoted tubular cell proliferation in IRI mice

**Figure 5:**
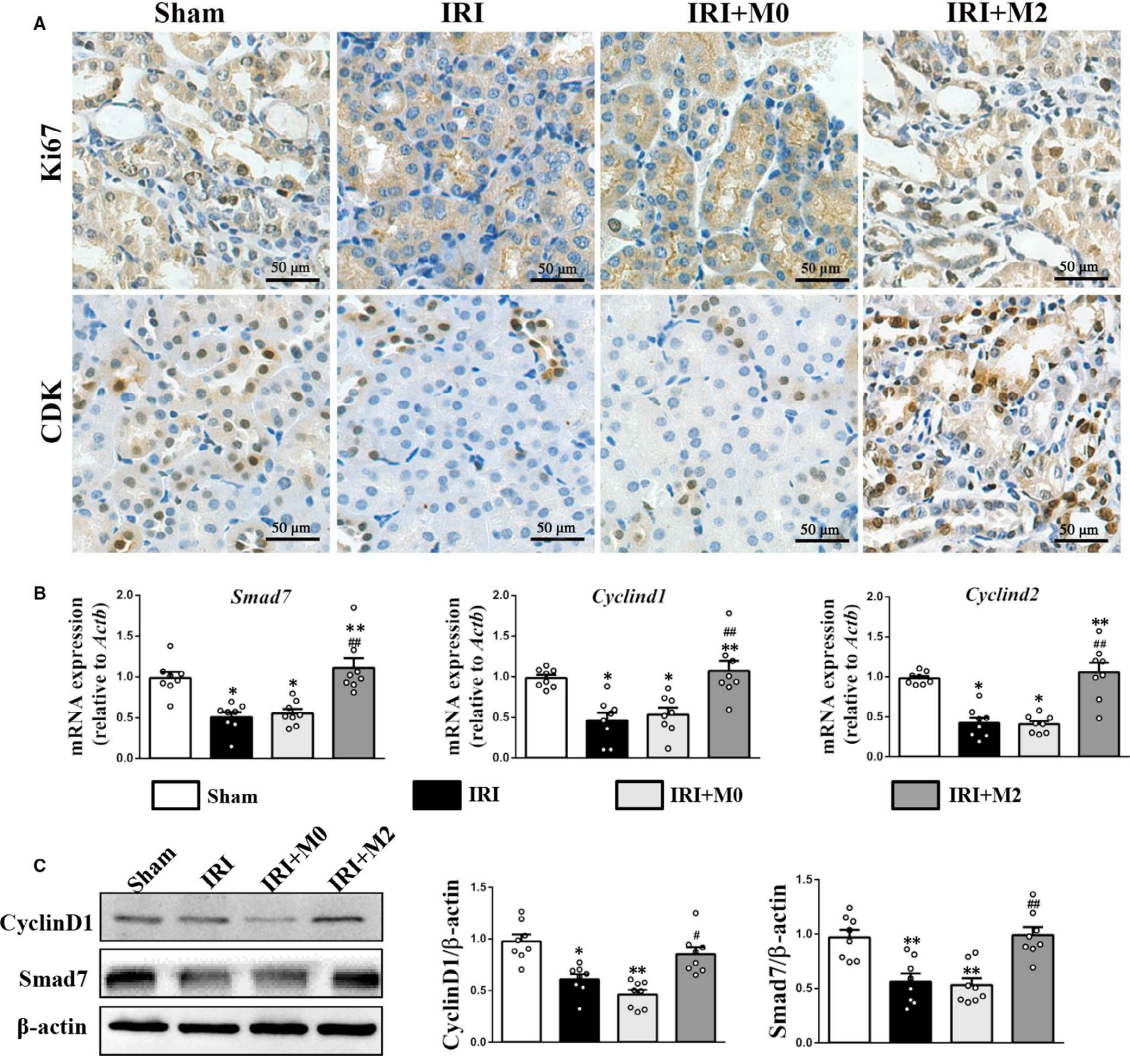
M2 macrophages promote tubular cell proliferation in kidneys after IRI. A, IHC staining for Ki67 and CDK at day 3 after IRI (Scale bar = 50 µm). B, Real‐time PCR analysis of Smad7, Cyclind1 and Cyclind2 mRNA level at day 3 after IRI. C, Western blot and quantification analysis of CyclinD1 and Smad7 protein level at day 3 after IRI. *P < .05, **P < .01 vs Sham. #*P* < .05, ##*P* < .01 vs IRI (n = 8)

The effect of M2 peritoneal macrophages on tubular epithelial cell proliferation after IRI was also analysed. The IHC staining analysis revealed that the levels of Ki67 and cyclin‐dependent kinases (CDKs) in the kidneys of the IRI + M2 group were higher than those in the kidney of sham, IRI and IRI + M0 groups (Figure 5A). Additionally, the IRI + M2 group exhibited higher protein and mRNA levels of CyclinD1 and CyclinD2 than the IRI and IRI + M0 groups (Figure 5B‐C). The original Western blot images are shown in Figure S2B.

### 3.5 Peritoneal M2 macrophages decreased cell apoptosis and expression of inflammatory factors in the PTECs under H/R conditions

**Figure 6:**
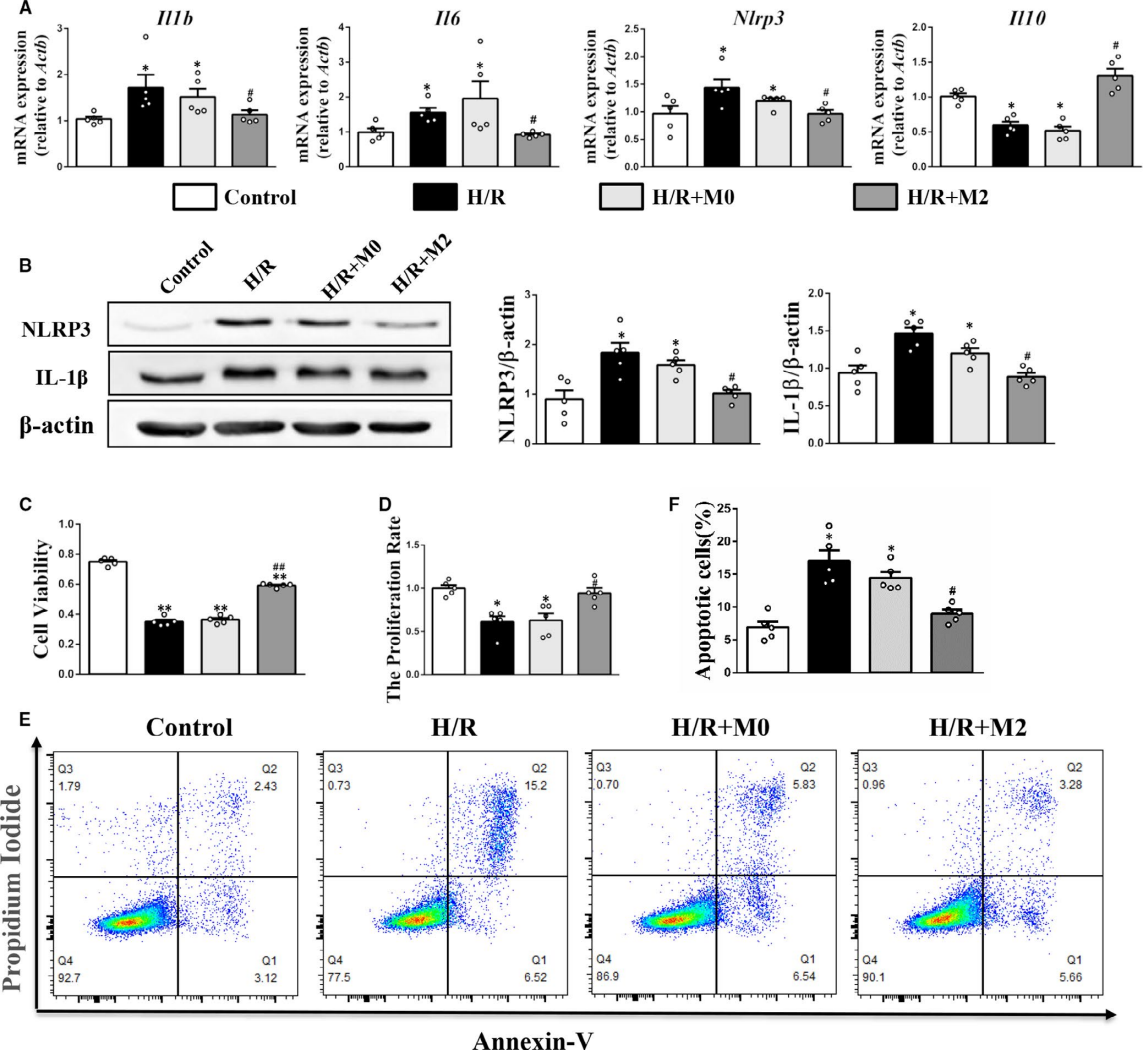
Effects of M2 macrophages on cell viability and inflammation in PTECs under H/R. A, Real‐time PCR analysis of Il1b, Il6, Nlrp3 and Il10 mRNA level. B, Western blot analysis of NLRP3 and IL‐1β protein level. (C) Cell viability was determined using the CCK‐8 assay. D, Cell proliferation rates detected by flow cytometry. E and F, The apoptotic ratios of PTECs determined by flow cytometry. *P < .05, **P < .01 vs Control. #*P* < .05, ##*P* < .01 vs H/R (n = 5)

The PTECs were isolated from the mouse renal cortex, and more than 95% of cells were positive for the epithelial marker (CK18) compared with the unstained control (Figure S1A‐C). The cell viability of PTECs decreased significantly under H/R conditions, which improved upon co‐culturing with M2 macrophages (Figure 6C). This was consistent with the cell proliferation rates determined by cell cycle assay (Figure 6D). The qPCR analysis revealed that the M2 macrophages inhibited *Il1b*, *Il6* and *Nlrp3* mRNA expression and promoted *Il10* mRNA expression in the PTECs under H/R conditions (Figure 6A). The Western blotting analysis revealed that the M2 macrophages exhibited decreased NLRP3 and IL‐1β protein expression in the PTECs under H/R conditions (Figure 6B). The original images of Western blots are shown in Figure S2C. Moreover, co‐culturing the PTECs with M2 macrophages decreased the cell apoptosis rates in the PTECs under H/R conditions (Figure 6E‐F). The flow cytometry gating strategy is shown in Figure S3C. The M0 macrophages had no effect on cell apoptosis and expression of inflammatory factors in the PTECs under H/R conditions (Figure 6A‐F).

### 3.6 Peritoneal M2 macrophages promoted PTEC proliferation by activating the TGF‐β/Smad7 pathway

**Figure 7:**
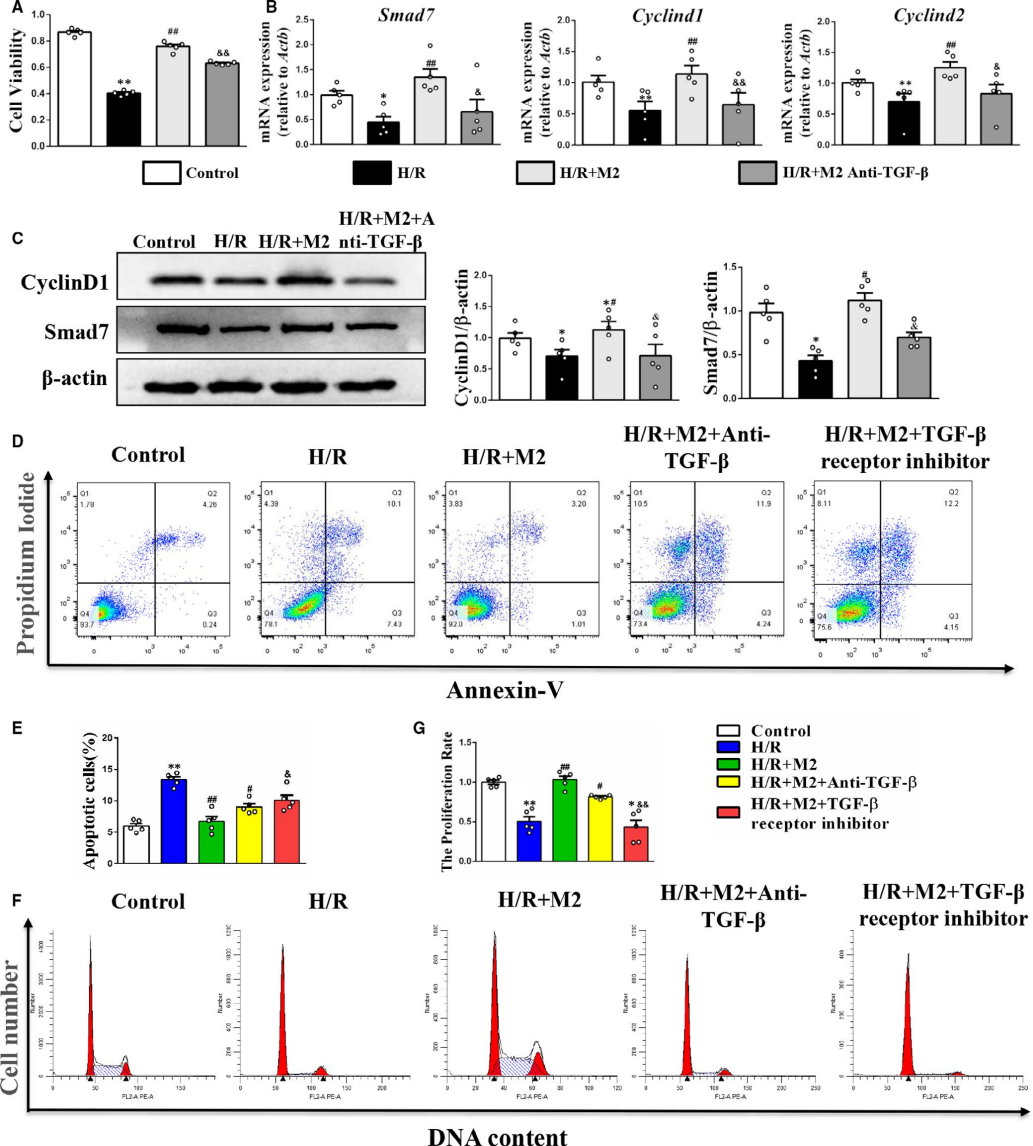
Effects of M2 macrophages on cell proliferation in PTECs under H/R. A, Cell viability was determined using the CCK‐8 assay. B, Real‐time PCR analysis of Smad7, Cyclind1 and Cyclind2 mRNA level. C, Western blot and quantification analysis of CyclinD1 and Smad7 protein level. D and E, The apoptotic ratios of PTECs were determined by flow cytometry. F and G, Cell proliferation rates were detected by flow cytometry. *P < .05, **P < .01 vs Control. #*P* < .05, ##*P* < .01 vs H/R. &*P* < .05, &&*P* < .01 vs H/R + M2 (n = 5)

The M2 macrophage transplantation could mitigate the decreased cell viability and proliferation index in the PTECs under H/R conditions. However, this proliferative effect was inhibited after treatment with anti‐TGF‐β (2 µg/mL, GeneTex) or TGF‐β receptor inhibitor (10 µmol/L, Selleckchem) (Figure 7A and D‐G). The flow cytometry gating strategy is shown in Figure S3D. The qPCR and Western blotting analyses revealed that the M2 macrophages promoted the mRNA and protein expression of Smad7, CyclinD1 and CyclinD2 in the PTECs under H/R conditions, which was significantly mitigated with the addition of anti‐TGF‐β (Figure 7B‐C). The original images of Western blots are shown in Figure S2D. Moreover, the inhibitory effect of M2 macrophages on cell apoptosis was also neutralized after treatment with anti‐TGF‐β or TGF‐β receptor inhibitor (Figure 7D‐E). The flow cytometry gating strategy is shown in Figure S3C.

## 4 DISCUSSION

Despite the progress in renal replacement strategies, there are increased incidences of AKI and the mortality rate among patients with AKI remains high.^4^ The macrophages are a key component of the innate immune system and play an important role in the initiation and progression of renal diseases.^31^, ^32^, ^33^, ^34^ In this study, we established a PD mouse model to collect the peritoneal mononuclear cells and evaluated the protective effect of peritoneal M2 macrophage transplantation on AKI in vivo and in vitro. Our results indicated that the peritoneal M2 macrophage transplantation significantly reduced the inflammatory infiltrates and promoted the proliferation of renal tubular epithelial cells after AKI.

Previous studies have evaluated the therapeutic potential of macrophages. Autologous macrophages were used in a phase I human trial to treat spinal cord injury. The study revealed that autologous macrophages promote neurological recovery.^35^ The regulatory macrophages induced by M‐CSF and interferon (IFN)‐γ were distinct from the conventional M2 phenotype, which could prolong the survival of heart allograft in mice.^36^ Additionally, a clinical trial reported that these macrophages could suppress the T cells and consequently decrease the dose of immunosuppressive drugs required for kidney transplantation.^37^ Qi et al evaluated the therapeutic potential of adoptive transfer of macrophages to treat kidney diseases and demonstrated the protective effects of different subsets of M2 macrophages and different sources of M2 macrophages in the experimental kidney disease models.^31^ The study also explored the feasibility and efficacy of monocytes/macrophages from peritoneal dialysate of patients and mice. The adoptive transfer of peritoneal M2 macrophages derived from peritoneal dialysate of mice decreased renal inflammation and injury in mice with AN.^31^ In this study, we demonstrated that the transplantation of peritoneal M2 macrophages derived from peritoneal dialysate of mice reduced renal inflammation and promoted tubular epithelial cell proliferation in IRI mice. These results suggested that the macrophages derived from peritoneal dialysate have potential therapeutic applications.

Inflammation is reported to be a key factor in the pathogenesis of AKI.^2^ The macrophages are one of the major immune cells involved in inflammation. The M2 macrophages are generally activated at a later phase after the initial injury. In the ischaemic AKI model, enhanced expression of M2 markers was observed in the kidney after 3 days with peak expression observed after 7 days.^38^, ^39^, ^40^ The M2 macrophages can be activated by M‐CSF, IL‐4, IL‐10, IL‐13 and TGF‐β. The alternatively activated M2 macrophages have a diverse functional repertoire, contributing to wound healing, fibrosis, insulin sensitivity and immunosuppression.^41^, ^42^, ^43^, ^44^, ^45^, ^46^, ^47^ The M2 macrophages are reported to promote tissue repair and remodelling in viral myocarditis and acute nephritis through secretion of various anti‐inflammatory mediators, such as IL‐4 and IL‐10.^42^, ^43^ Moreover, the peritoneal macrophages exhibited decreased secretion of TNF‐α, IL‐1β and IL‐6 when compared to the peripheral macrophages.^44^, ^46^ This suggested that the peritoneal macrophages have potential applications in cell therapy. The results of this study demonstrated that the peritoneal M2 macrophages could improve renal function, inhibit inflammatory cytokine expression and reduce T‐lymphocyte infiltration in the kidneys of IRI mice. Additionally, the peritoneal M2 macrophages suppressed the expression of inflammatory factors and inhibited cell apoptosis in the PTECs under H/R conditions. This indicated that the peritoneal M2 macrophages partially ameliorated IRI‐AKI via direct anti‐inflammatory and anti‐apoptotic effects.

Renal tubular cell damage is an important mechanism underlying the pathogenesis of AKI.^44^ Several studies have demonstrated that the macrophages play a reparative role during the recovery phase of AKI.^45^, ^46^, ^47^ The depletion of macrophages during renal repair was associated with sustained renal inflammation and impaired tubular regeneration.^48^ However, the mechanisms underlying the promotion of PTEC proliferation by the M2 macrophages in the post‐ischaemic kidney are not well understood. The M2 macrophages are reported to promote cell proliferation by up‐regulating Smad7 expression in the beta‐cells.^49^ Consistently, we observed significantly enhanced expression of Smad7, a TGF‐β superfamily signalling inhibitor, in the renal tubular cells after AKI. Smad7 is reported to directly up‐regulate the cell cycle activators, such as CyclinD1 and CyclinD2, which consequently promote beta‐cell proliferation.^50^ To further elucidate the underlying mechanism, we used an in vitro transwell system to co‐culture the peritoneal macrophages and PTECs. Our results demonstrated that the M2 macrophages but not M0 macrophages were sufficient to induce Smad7 expression and consequently promote PTEC proliferation. The inhibition of the TGF‐β/Smad7 signalling pathway using antibody or TGF‐β receptor inhibitor markedly inhibited the PTEC proliferation under H/R conditions. Although the M2 macrophages are reported to be involved in tissue repair by promoting neovascularization and cell growth,^51^ the results of this study indicated that they directly promote PTEC proliferation after AKI.

The peritoneal monocytes/macrophages derived from peritoneal dialysate are a rich source of M2 macrophages than can be used for cell therapy. Moreover, these cells can be polarized into different phenotypes for targeting different diseases. In this study, the adoptive transfer of peritoneal M2 macrophages mitigated renal dysfunction and inflammation after AKI. Additionally, the peritoneal M2 macrophages directly promoted the proliferation of renal tubular epithelial cells through the TGF‐β/Smad7 signalling pathway. Therefore, the findings of this study indicated that autologous peritoneal macrophage‐based cell therapy can be a potential treatment for kidney disease and other immunological and inflammatory diseases. Future studies must focus on evaluating the therapeutic potential of allogeneic peritoneal mononuclear cell‐derived M2 macrophages for kidney injury.

## 5 CONCLUSION

Our study demonstrated that IRI induces severe renal inflammation, cell apoptosis and tubular epithelial cell damage. Treatment with the peritoneal M2 macrophages effectively mitigated the renal injury in AKI mice. The peritoneal M2 macrophages secrete TGF‐β and IL‐10 to ameliorate renal inflammatory responses. Moreover, the peritoneal M2 macrophages activated the TGF‐β/Smad7 pathway to promote tubular epithelial cell proliferation via up‐regulation of the cell cycle activators, CyclinD1 and CyclinD2. This study provided insights into the complex events involved in AKI and indicated that the peritoneal M2 macrophages can be potentially used for cell therapy for renal diseases.

## CONFLICT OF INTEREST

The authors declare that they have no competing interests.

## AUTHOR CONTRIBUTIONS

All authors contributed extensively to the work presented in this paper. CSW, YRL and JPL designed the project. RWM, CSW, FPZ, MZ, LSY, GNL and LL performed the experiments and analysed statistical data. CSW and RWM wrote the manuscript. YNC, JQC, JPL and YRL provided critical suggestions revised the manuscript. All authors have read and approved the final manuscript.

## Data Availability

All data generated or analysed during this study are included in this published article.
