# Supplementary material for: Peritoneal M2 macrophage transplantation as a potential cell therapy for enhancing renal repair in acute kidney injury
Source: J Cell Mol Med. 2020 Jan 31;24(6):3314–27. doi: 10.1111/jcmm.15005 (PMC7131941; doi:10.1111/jcmm.15005)
Supplement: Supplementary file 1 [file JCMM-24-3314-s001.docx]

**Table S1. Primers used for qPCR analysis**

| **Gene Symbol** | **Species** | **Primer sequence 5ˊto 3ˊ** |
| --- | --- | --- |
| *Mr* | Mouse | F: GTCTGAGTGTACGCAGTGGTTGG R: TCTGATGATGGACTTCCTGGTAGCC |
| *Il10* | Mouse | F: GCTCTTACTGACTGGCATGAG R: CGCAGCTCTAGGAGCATGTG |
| *Tgfb1* | Mouse | F: CAACAATTCCTGGCGTTACCTTGG R: GAAAGCCCTGTATTCCGTCTCCTT |
| *Arg1* | Mouse | F: TGCTCACACTGACATCAACACTCC  R: TCTACGTCTCGCAAGCCAATGTAC |
| *Ccl17* | Mouse | F: GAGCCATTCCCCTTAGAAAG R: AGGCTTCAAGACCTCTCAAG |
| *Il1b* | Mouse | F: CACCTCTCAAGCAGAGCACAG R: GGGTTCCATGGTGAAGTCAAC |
| *Il6* | Mouse | F: GTTCTCTGGGAAATCGTGGA  R: TGTACTCCAGGTAGCTATGG |
| *Tnfα* | Mouse | F: CCAGGAGAAAGTCAGCCTCCT R: TCATACCAGGGCTTGAGCTCA |
| *Nlrp3* | Mouse | F: TCAACAGTCGCTACACGCAG R: CCTCTCGGCAGTGGATAAAG |
| *Smad7* | Mouse | F: CTGTGTTGCTGTGAATCTTACG  R: GAGACTCTAGTTCACAGAGTCG |
| *Cyclind1* | Mouse | F: CGTATCTTACTTCAAGTGCGTG R: ATGGTCTCCTTCATCTTAGAGG |
| *Cyclind2* | Mouse | F: GTCTGTGAGGAACAAAAGTGTG R: CTGAAGATGGGTCTTAGGAGTC |
| *Actb* | Mouse | F: CGTGCGTGACATCAAAGAGAA R: AACCGCTCGTTGCCAATAGT |
|  |  |  |

**Fig. S1**

**
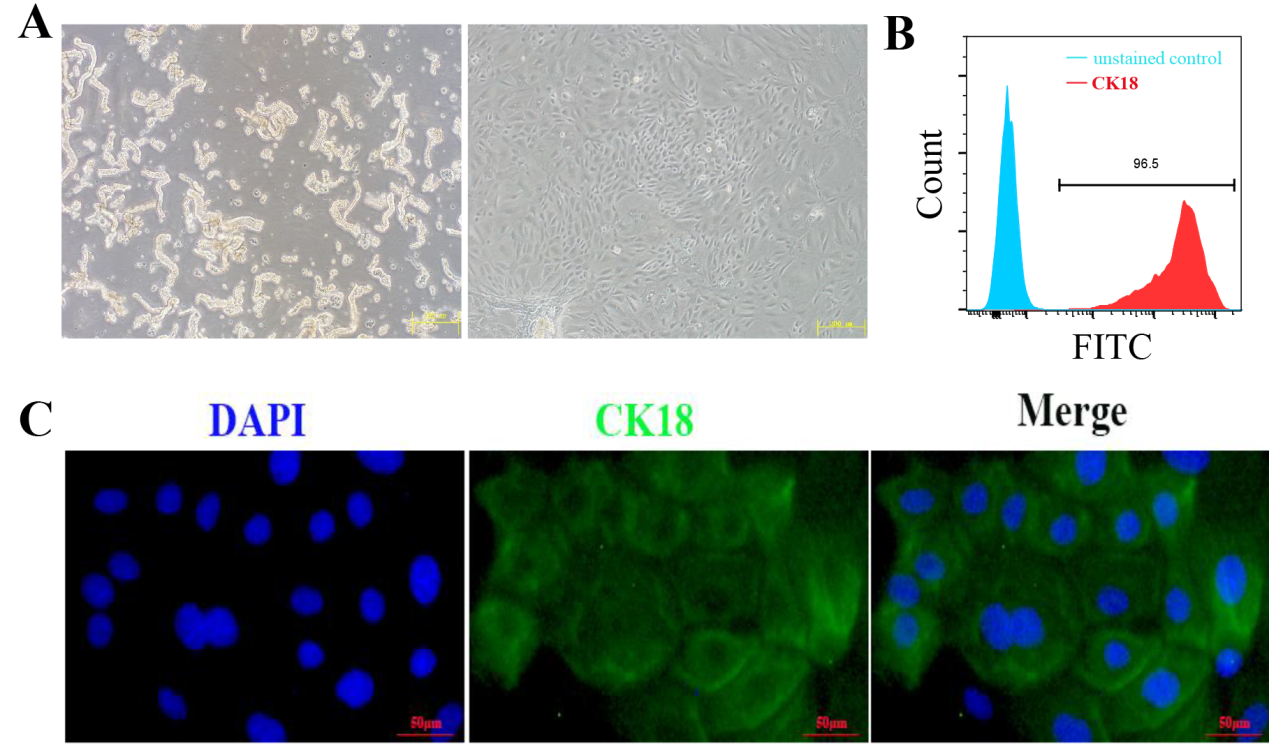
**

**Fig. S1 Isolation and validation of mouse** **primary renal tubular epithelial cells(PTECs).**

(A) Microscopic views of PTECs isolated from mouse renal cortex from fresh kidney. Left: freshly purified renal tubules. Right: cultured renal tubule epithelial cells after 7 days (Scale bar = 200 μm). (B) Flow cytometric analysis of CK18 in mouse PTECs isolated from mouse renal cortex, blue coloured data represents a unstained control, red cooured data represents a CK18 positive proportion. (C) Immunofluorescence staining analysis of CK18 in mouse PTECs isolated from mouse renal cortex.

**Fig. S2**


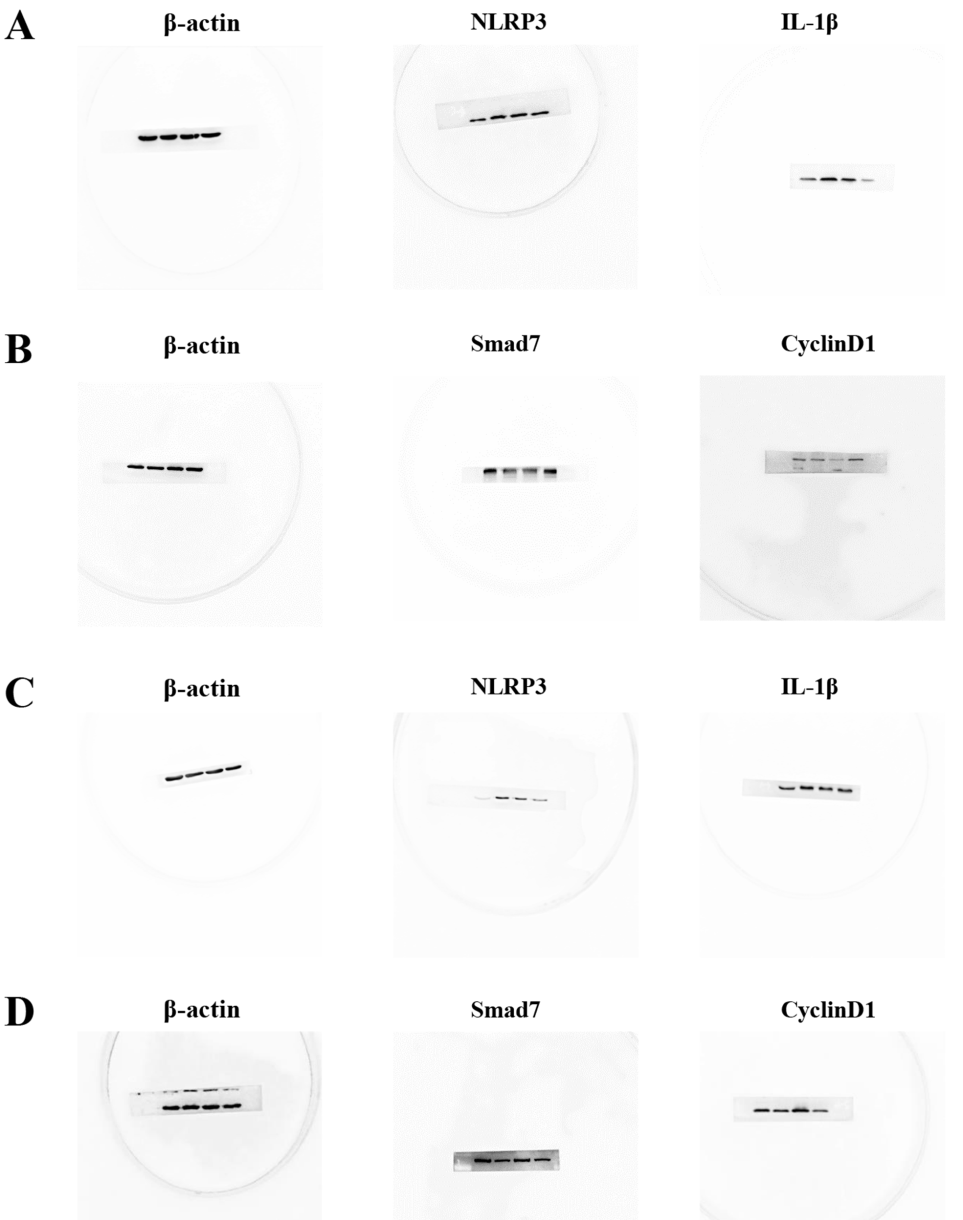


**Fig. S2. Raw data of the original unedited gel images.**

(A) Unedited western blot figures for Figure 3D. (B) Unedited western blot figures for Figure 5C. (C) Unedited western blot figures for Figure 6B. (D) Unedited western blot figures for Figure 7C.

**Fig. S3**


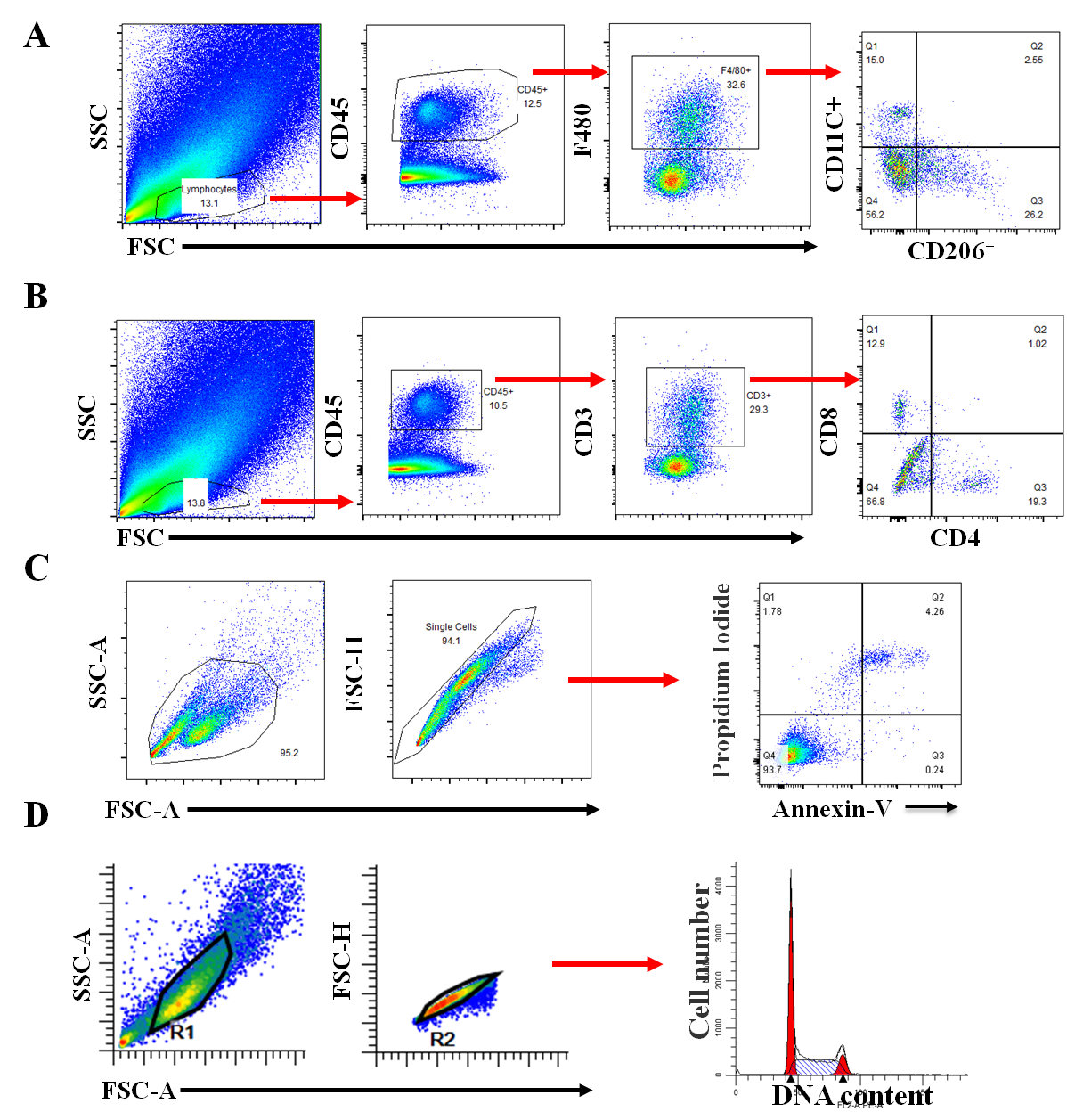


**Fig. S3 Representative flow cytometry gating strategy .**

(A) Flow cytometry gating strategy for Figure 3A. (B) Flow cytometry gating strategy for Figure 3F. (C) Flow cytometry gating strategy for Figure 6E and 7D. (D) Flow cytometry gating strategy for Figure 7F.

**Fig. S4**

**
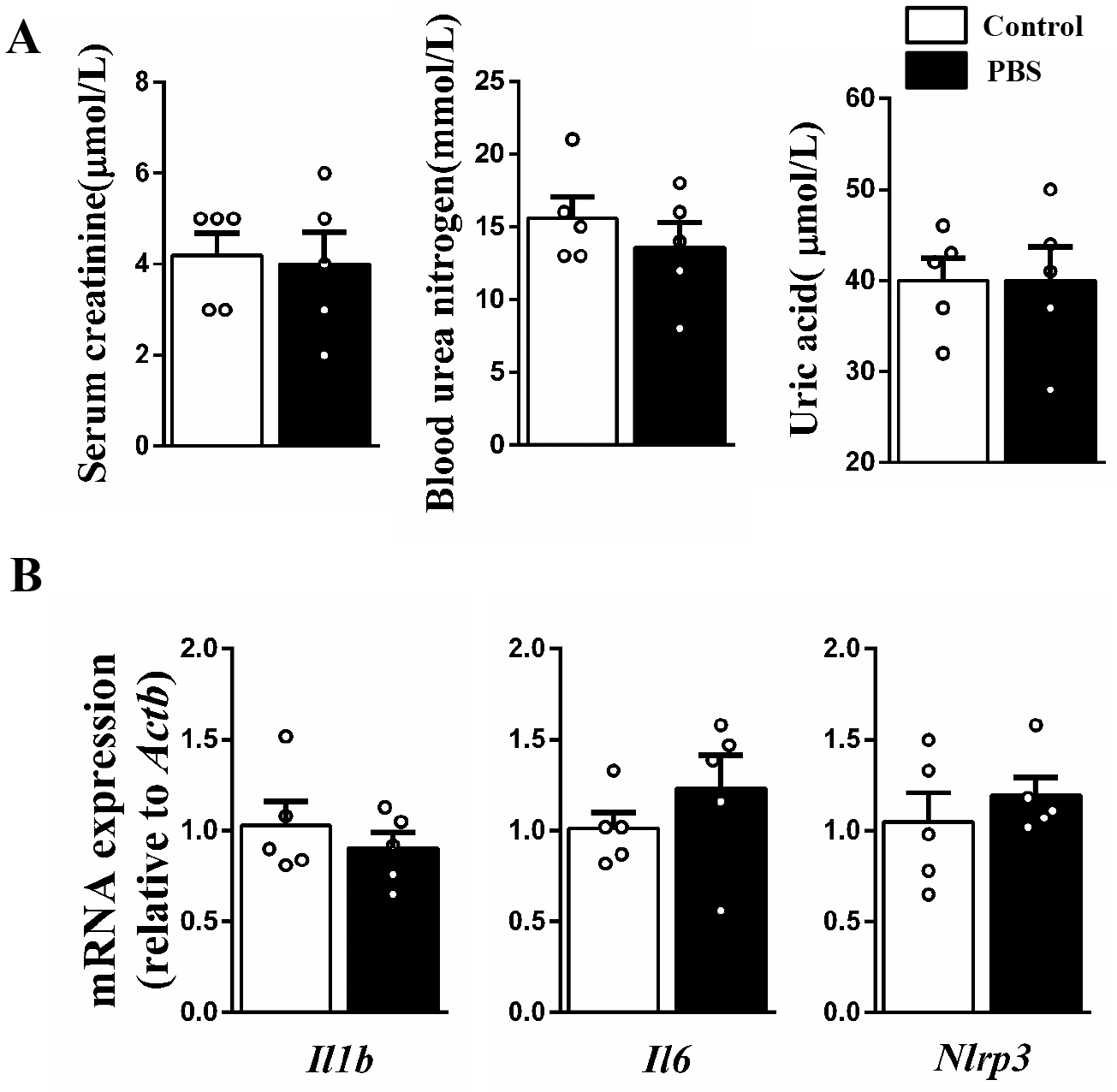
**

**Fig. S4 PBS injection has no obvious damage to the kidney.**

(A) Serum Crea, BUN and uric acid (UA) 3 days after PBS injection (n=5). (B )Real-time PCR analysis of kidney *Il1b*, *Il6* and *Nlrp3* mRNA level 3 days after PBS injection (n=5).
